# Social exclusion leads to attentional bias to emotional social information: Evidence from eye movement

**DOI:** 10.1371/journal.pone.0186313

**Published:** 2017-10-17

**Authors:** Zhuohao Chen, Jinchen Du, Min Xiang, Yan Zhang, Shuyue Zhang

**Affiliations:** 1 School of Education, Guangxi University, Nanning, Guangxi Zhuang Autonomous, China; 2 School of Stomatology, Wenzhou Medical University, Wenzhou, Zhejiang, China; 3 Department of Educational Psychology, The Chinese University of Hong Kong, Hong Kong, China; Leiden University, NETHERLANDS

## Abstract

Social exclusion has many effects on individuals, including the increased need to belong and elevated sensitivity to social information. Using a self-reporting method, and an eye-tracking technique, this study explored people’s need to belong and attentional bias towards the socio-emotional information (pictures of positive and negative facial expressions compared to those of emotionally-neutral expressions) after experiencing a brief episode of social exclusion. We found that: (1) socially-excluded individuals reported higher negative emotions, lower positive emotions, and stronger need to belong than those who were not socially excluded; (2) compared to a control condition, social exclusion caused a longer response time to probe dots after viewing positive or negative face images; (3) social exclusion resulted in a higher frequency ratio of first attentional fixation on both positive and negative emotional facial pictures (but not on the neutral pictures) than the control condition; (4) in the social exclusion condition, participants showed shorter first fixation latency and longer first fixation duration to positive pictures than neutral ones but this effect was not observed for negative pictures; (5) participants who experienced social exclusion also showed longer gazing duration on the positive pictures than those who did not; although group differences also existed for the negative pictures, the gaze duration bias from both groups showed no difference from chance. This study demonstrated the emotional response to social exclusion as well as characterising multiple eye-movement indicators of attentional bias after experiencing social exclusion.

## Introduction

Social exclusion occurs when individuals are isolated, disregarded, or rejected in social interactions [1]. Social exclusion can be experienced in many occasions, including a refusal of an offer, dismissal from a job, rejection from colleagues, or exclusion from games among children [2]. Previous studies suggest that social exclusion can even affect people’s emotional responses but evidence based on behavioural indictors remains limited. Therefore, this study combined the use of a self-reporting method and an eye-tracking technique to reveal changes in belongingness needs of people and characterise behavioural patterns of attentional bias towards socio-emotional stimuli after social exclusion.

### Social exclusion and the consequences

Social exclusion reflects interpersonal dissonance. Being conceptually opposed to social inclusion, social exclusion depicts the phenomenon of individuals being rejected by social groups or other individuals in social interactions such that they cannot build social relationships and satisfy their belongingness needs (*e*.*g*.[3]). This rejection could exist in various domains (*e*.*g*., work vs. private relationships), among different groups (*e*.*g*., majority vs. minority groups), and in different forms (*e*.*g*., explicit vs. implicit). Social exclusion occurring under different conditions communicates the same message: the victim is disliked, disagreed with, unpopular, or even hated. This information could be difficult for the victim to accept, and might cause painful experiences for those individuals being socially excluded [3,4].

Social exclusion causes cognitive, emotional, and behavioural consequences in terms of interpersonal connection. In cognition, people make negative interpersonal appraisals of those who conduct social exclusion behaviours [5,6] and positively appraise those who might contribute to their relational resources, which reflects a motive for interpersonal reconnection.[6]. In emotional terms, social exclusion is associated with negative affect, such as loneliness, jealousy, anxiety, or depression [7,8]. Indeed, a three-year follow-up study among a group of youths supported the conclusion that social exclusion causes depression [9]. Other emotional responses, such as grief, sadness, and indignation, were also observed [5]. Social rejection by people of different races causes more anger [10]. In terms of behaviour, social exclusion might undermine people’s self-regulation ability [11] and evoke aggressive behaviour and attacks from the victim, especially toward those who exclude the victim [12]. These findings imply mental tension in the social interactions of socially excluded individuals.

Being rejected by others might elevate the need to belong. Social exclusion causes lowered self-esteem, depression, sadness and loneliness[5,6,8], while belongingness brings security, warmth, and cheerfulness. The link was stated more specifically in the need-threat theory of ostracism [13]. The theory developed a model in which different conceptualizations of ostracism were unified, and more importantly, it noted that the four fundamental needs including the need to belong, were threatened after ostracism. Individuals show higher social susceptibility and seek to increase their sense of belonging especially after being socially excluded[14,15], even in the context of the internet [16]. The motivation of gaining a stronger sense of belongingness might appear in various forms, such as working harder as member of a team [17] and being more likely to listen to others [16]. In this study, we propose that the need to belong after social exclusion might be accompanied by certain primary behaviours such as an attentional bias toward socio-emotional information.

### Attentional bias after social exclusion

Attentional bias refers to a condition in which individuals distribute attention to different aspects of neutral and threatening stimuli to varying degrees[18–20]. According to earlier research [21], attentional bias can appear in two forms: 1) attentional vigilance or attentional facilitation, in which attention is attracted faster, or more easily, by certain stimuli or information, and 2) attentional avoidance, in which individuals avoid paying attention to certain stimuli. The development of eye-tracking techniques has helped to define and measure a third characteristic–difficulty in disengaging attention (also referred to as attentional fixation)–which causes individuals difficulty in transferring their attention from certain stimuli [22,23].

Former studies show that attentional bias might correspond to specific psychological states. That is, when individuals hold a motive or a certain need, they are more likely to favor social information that might fulfill their needs. For example, a state of low self-esteem contributes to more interference on rejection words but less on acceptance words in a “Rejection Stroop task” [24], and highly narcissistic individuals have difficulty disengaging from success words [25]. Regarding the lack of a sense of belonging, in this study, we argue that individuals are motivated to seek to fulfill the need to belong and are probably more sensitive to social cues that imply social acceptance. Thus, attentional bias might appear after social exclusion. Earlier research has explored this issue. DeWall and colleagues [26] used feedback indicating a “future alone” and direct interpersonal rejection to induce social exclusion in different experiments. They then found a stronger sensitivity to searching for emotional faces in a crowd of neutral faces (Experiment 1), more attentional fixation on smiling faces (Experiment 2 and 3), and slower disengagement of attention from smiling faces (Experiment 4).

Limitations in the studies of DeWall and colleagues[26] have encouraged the current study. First, they use the “future alone” paradigm in the first three experiments, which might be not sufficient to establish a sense of social exclusion. “Future alone” could be a natural result of people growing old and losing the people around them, but being socially excluded could be an experience caused by others, along with the victim’s anger and other responses. Second, although a direct social rejection manipulation was used in Experiment 4, only disengagement from social cues was examined in that study. The conclusion was that socially excluded individuals showed significantly higher fixation on smiling faces than non-excluded people, but no difference was observed regarding fixation on the negative faces. However, they did not test early-stage attentional vigilance or avoidance regarding the negative faces. Social exclusion, as it damages feelings of belongingness, might increase individuals’ avoidance tendency in response to negative faces. Hence, this study aims to explore attentional bias after social exclusion in a more systematic fashion.

### Overview of the current study

In this research, a pilot study tested the validity of a ball-tossing game in manipulating social exclusion, and the Positive and Negative Affect Schedule [27] was used as a manipulation check. The formal experiment used the ball-tossing game [13] to induce a sense of social exclusion and the dot-probe task combined with an eye-tracking technique [28] to explore attentional bias after social exclusion. We also measured the need to belong in the formal experiment after social exclusion manipulation but before attentional bias measurement. In fact, social exclusion could elevate individuals’ sensitivity to social cues. This sensitivity could both go with an increased vigilance toward threatening social cues, and a preference toward positive social information. In this case, we posited that individuals might be vigilant to both negative and positive social cues, but show more fixation on positive cues as they seek positive social information after social exclusion. Specifically, we hypothesise that:

1. Socially excluded individuals report higher negative but lower positive affect and stronger needs of belongingness compared with those being not excluded.
2. Socially excluded individuals perform attentional bias toward positive facial affect rather than neutral one.
3. Socially excluded individuals might show early-stage attentional bias (e.g., directional bias) toward the negative facial affect in paired negative-neutral facial pictures.
4. Socially excluded individuals might show longer fixation bias (e.g., gaze duration bias) toward positive pictures but not negative pictures, as they seek belongingness after social exclusion.

### Pilot study

The pilot study aims to validate the operational effectiveness of the social exclusion paradigm: the ball-tossing game. Its validity is tested using the PANAS.

## Methods

### Participants

A total of 12 college students (6 males and 6 females) from Guangxi University, mainland China, joined the experiment that ran from 1 January, 2016 to 5 January, 2016. The age range was 18 to 28. They were invited to participate in the study when walking within the campus of the university, China.

### Materials

#### Positive and negative affect schedule

Affective responses are often used to measure the operational effect of social exclusion [26]. In the present study, the PANAS from Watson and colleagues [27] measured the participants’ emotional reactions after social exclusion (for another instrument of manipulation check, the need threat scale, see van Beest & Williams[29]). The PANAS can be divided into positive and negative affect subscales, each containing nine items measured on a five-point Likert scale (1 = *not at all*, 5 = *very strong*). Higher scores indicate stronger affect. Previous studies indicate good reliability of both sub-scales [27].

#### Procedures

**Fig 1:**
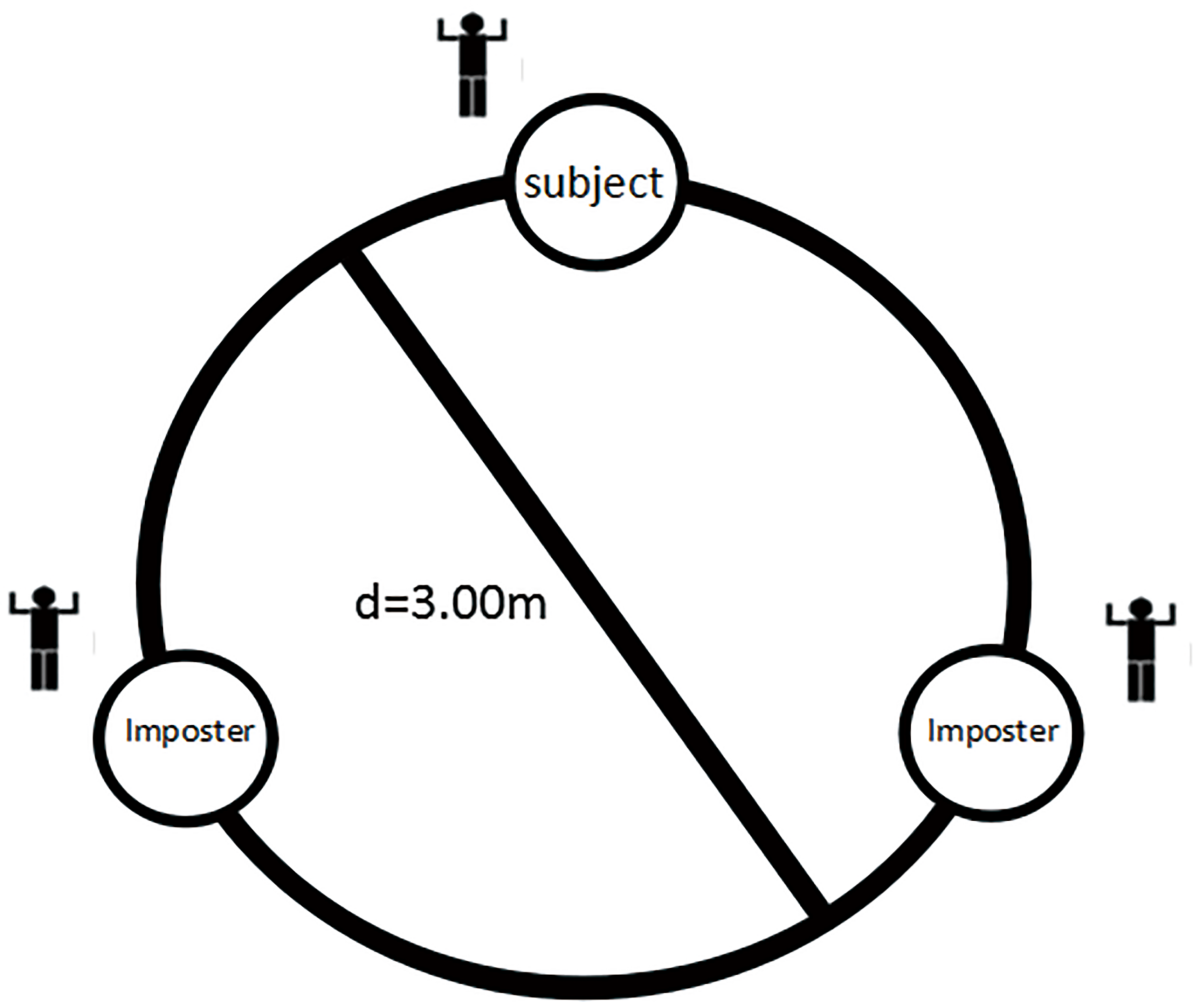
Layout of experimental site—subjects and imposters. Note: Three people stand, equidistant from each other, on the edge of a circle (diameter *D*, 3 m).

All the participants gave informed written consent to the experimental procedures before the study, which were approved by the Guangxi University Institutional Ethical Review Board. The ball-tossing game this study used was modified from Williams [13]. The participants were randomly divided into the social exclusion group and the control group. A confederate was arranged, in advance, to wait at the laboratory, and another confederate entered one minute after each participant arrived. All three were of the same gender. The participants were informed that the experiment was related to sports psychology. Before the formal experiment, they first participated in a simple ball-tossing game and were, respectively, assigned to three equidistant circles (*D*: 3 m, as shown in Fig 1). Subsequently, they were required to complete a continuous 2-minute pass, starting from any one of them. The server might pass the ball to one of the other two, and then the catcher could pass it to one of the other two, in which way the cycle continued. Neither foot movement nor the ball being falling to the ground was allowed while passing. The passing began after a few minutes of free practice, and the experimenter monitored the time in another room. Two minutes later, the experimenter ordered a halt. In the first half-minute of the two minutes, the three persons balanced the passing and made eye contact with each other; in the second half-minute, the confederate passed the ball to the participant less frequently, and the participant could receive the ball only two or three times; and in the last minute, the two confederates passed to each other while maintaining eye contact, and the participant could not receive the ball or make eye contact with the confederates. After the game, the participants completed the PANAS. Before they left, the participants were required to describe their experience in playing the ball-tossing game and were later debriefed about the aim of the game and were thanked with small gifts. All of the participants in the control group completed the PANAS without playing the ball-tossing game.

## Results and discussions

A *t*-test checked the difference between the social exclusion group and the control group in both positive and negative affect. The results showed that the score of the excluded group on the positive affect subscale (*M* = 20.50 ± 5.61) was significantly lower than that of the control group (*M* = 29.33 ± 7.84), *t* (10) = 2.24, *p* < 0.05, indicating that the socially excluded participants experienced lower positive emotions. On the negative affect subscale, the socially excluded group (*M* = 29.00 ± 8.58) scored significantly higher than the control group (*M* = 17.67 ± 6.19), *t* (10) = –2.63, *p* < 0.05, showing that the socially excluded participants experienced stronger negative affect.

The results supported the efficiency and reliability of the ball-tossing game in generating a sense of social exclusion; therefore, the paradigm was adopted for the formal experiment.

### The formal experiment

The formal experiment aims to investigate the attentional bias of socially excluded individuals regarding non-verbal social inclusion/rejection information stimuli. This study verified whether individuals would become more sensitive to affect and perform attentional bias after social exclusion. The characteristics of their cognitive processes were analysed via the eye-tracking technique. Meanwhile, we investigated whether there were differences in attentional bias and cognitive processes related to positive and negative facial affect between the individuals being excluded and those not being excluded affect.

## Methods

### Participants

A total of 46 college students, including 20 males and 26 females from mainland China, joined in the experiment which ran between 5 January, 2016, and 31 January, 2016. They were invited to participate in the study when walking within the campus of Guangxi University, China. The age range was 18 to 28. Among them, 21 participants were randomly assigned to the social exclusion condition while the rest were assigned to the control group. Based on the standards of data processing (see the data processing section), 38 participants (*M*_age_ = 21.81 ± 2.75) entered data analysis, including 17 (7 males and 10 females) from the social exclusion group and 21 (6 males and 15 females) from the control group.

### Materials

#### Facial affective pictures

Facial affective pictures were obtained from the Chinese Facial Affective Picture System [30]. A total of 20 pictures (half showing a female, half a male) were selected from the positive and negative affective libraries, respectively; at the same time, 80 pictures (same gender ratio) were chosen from the neutral affective library. In the end, 40 positive-neutral (P-O) affective pairs, 40 negative-neutral (N-O) affective pairs, and 40 neutral-neutral (O-O) affective pairs were formed. Faces of the same gender made a pair. Each pair appeared twice in the test, with one appearing on the left side and the other on the right side of the screen.

We included the neutral affective pairs mainly as a cover to prevent the participants from guessing the actual purpose of the study. A total of 24 additional facial affective pictures from the neutral affective library of the CFAPS formed 12 O-O affective pairs for practices before the formal task began.

#### The *PANAS*

The PANAS [27] measured the positive and negative affect after the ball-tossing game, as in the pilot study. A higher score indicated a stronger affect for both subscales.

#### The need to belong scale

The participants’ belongingness needs were measured by the 10-item Need-to-Belong Scale (NBS, from Leary, Herbst, & McCrary[31]) using a five-point scale (1 = *strongly disagree*, 5 = *strongly agree*). A higher score indicated a stronger need to belong. The scale showed high reliability, with Cronbach’s α = .80.

### Instruments

An Eyelink 1000 eye-tracking system (brand: SR Research) was used to collect the subjects’ eye-tracking data, with a sample rate of 1000 Hz and a mean viewing position error of < 0. 6°. The experimental stimuli were rendered on a 19-inch (0.48 m diagonal) screen. The refresh rate was 100 Hz, and the resolution was 1024 × 768 pixels. The distance from the participant’s eyes to the screen was 600 mm, and the distance between the eye-tracker camera and the screen was 70 mm.

### Procedures

All participants gave their informed, written, consent to the experimental procedures at the beginning, as approved by the Guangxi University Institutional Ethical Review Board. In this study, the participants were randomly assigned into the social exclusion group and the control group. In the social exclusion group, the participants first finished the ball-tossing task, during which they experienced social exclusion. Then, they finished the PANAS and the NBS. Thereafter, they moved to the probe detection task. They were informed that it was a task about spatial cognitive abilities. The participants were required to sit in front of the computers, keep their chins on the trays fixed in front of the screens, and follow the task procedures.

**Fig 2:**
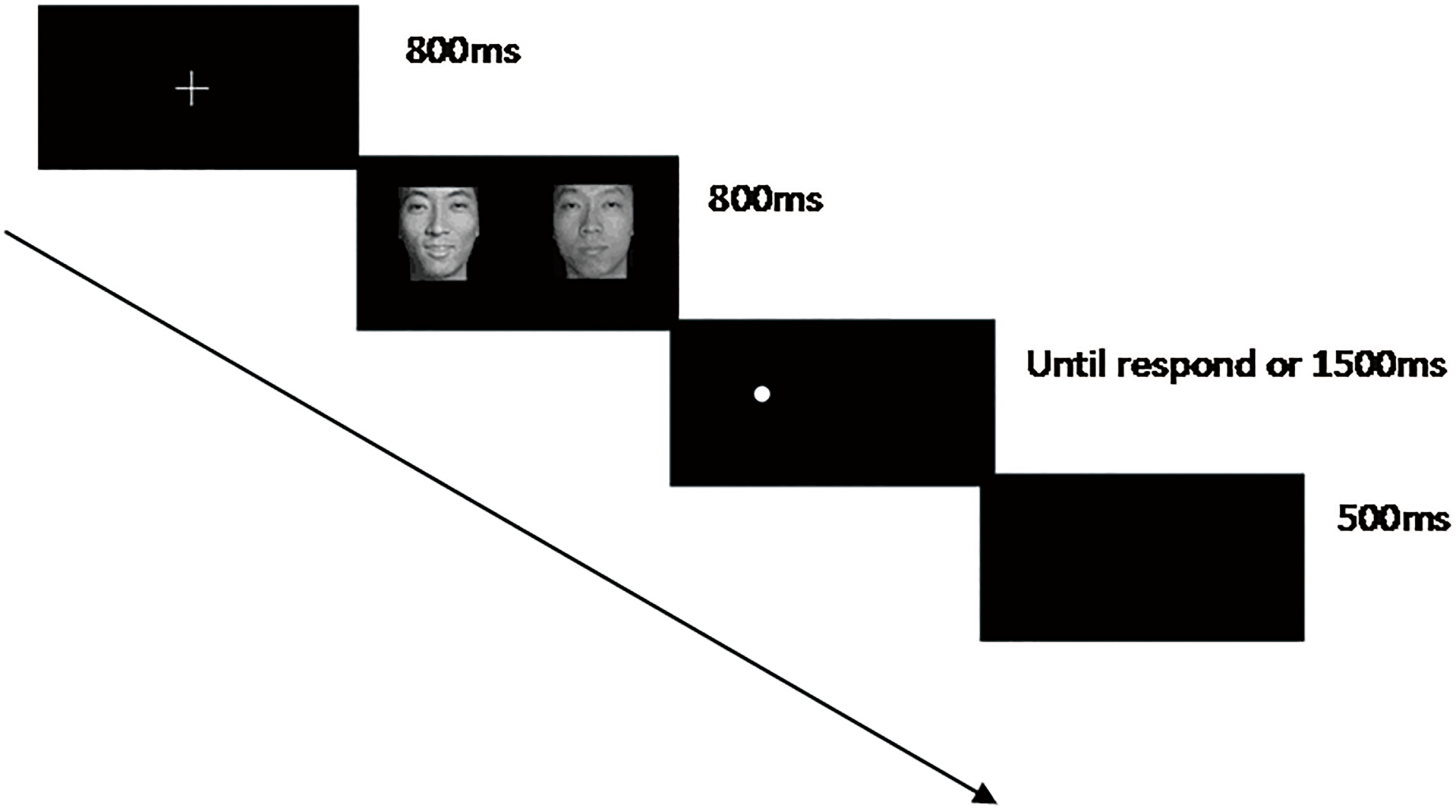
Probe detection task procedure (example of P-O affective pairs, with the probe dot and the facial affective picture on the same side). Note: Considering that the emotional faces used in the study contain private information, we use cartoon images of the emotional pictures instead of the real photographs in this figure (as with the other figures in this study).

The program first presented instructions about the aim and the procedures of the task. Upon confirmation that the participants fully understood the procedures, the program then executed the calibration program for the eye-tracking system. A nine-point calibration was adopted for this system. The calibration was validated after reaching the standard level (Successful), and the system formally entered the experiment after reaching the desired level (Good). The classical probe detection task [32] for the trial was modified for the current study, as shown in Fig 2. At the beginning, a 2 cm × 2 cm cross mark appeared at the centre of the white screen and remained there for 800 ms. The subjects were required to look attentively at the cross. Then, the facial affective picture appeared on either the right or the left side for another 800 ms (no action was needed upon the display of the pictures). Once the picture disappeared, a probe dot appeared randomly on the screen to the left or right of the cross mark, and the participants were required to quickly judge the position of the dot by pressing the corresponding key (left: ‘F’ and right: ‘J’) with the index fingers. Once a key had been pressed, the probe dot disappeared (or the dot automatically disappeared if there was no response within 1500 ms), and a blank black screen, lasting for 500 ms, followed. The next trial started after the blank screen.

The principle of this classical probe detection paradigm is that, when an individual concentrates on a visual area for a certain time, the time before their response to the probe stimuli near this area will be shorter than if the probe dot is farther away, or on the opposite side [33]. For example, if the subjects originally concentrate on the left side when the stimulus appears there, they should respond faster when the probe dot appears in the same position or on the same side but slower when it appears on the opposite side. We combined the procedure with an eye-tracking technique. In this way, we were able to detect changes in attentional bias before, and after, the occurrence of stimuli.

In the control group, the participants started the probe detection task without experiencing social exclusion. All eye fixation times and durations were recorded while the participants were going through the task.

The program included a total of two blocks, of which the first was a practice program that contained 12 trials designed merely to aid understanding of process (during the practice sessions, the O-O affective pairs appeared, and none of them appeared again in the formal experiment). The second block comprised was the formal experiment, consisting of 40 P-O trials, 40 N-O trials, and 40 O-O trials, with these 120 trials appearing randomly. The data record of each trial started from the presence of a small cross and lasted until the judgement process was ended by the participant. The program used in this study was edited using Experiment Builder, a programming software for Eyelink 1000 (brand: SR Research).

All participants were debriefed about the social exclusion game and the aim of the study, and were thanked with small gifts.

### Data preparation

#### Data and indicators

Based on previous findings [34,35], four eye-tracking indicators: first fixation directional bias, first fixation latency bias, first fixation duration bias, and gaze duration bias, are adopted as indicators for attentional bias in the present study. They are calculated by way of the following methods:

1. First fixation directional bias = number of times that the first fixation is on the facial affective pictures divided by the number of times that the first fixation is on all of the trials’ pictures. It indicates the initial directional attentional bias and reveals the attentional vigilance. A score greater than 50% indicates that a directional bias initially exists toward the facial affective pictures, while a score less than 50% means that a bias exists away from the facial affective picture. When a score statistically equals 50%, it indicates that no directional bias exists.
2. First fixation latency bias score = latency of the first fixation on the facial affective picture minus latency of the first fixation on the neutral picture. The objective is to test the speed of response to the probing stimuli, thereby reflecting attentional vigilance. A score greater than zero indicates a bias in slowly probing the facial affective picture, while a score less than zero reveals a bias in accelerating vigilance in probing the facial affective picture. When a score is statistically equal to zero, no bias exists.
3. First fixation duration bias = duration of first fixation on the facial affective picture minus duration of first fixation on the neutral picture. A score greater than zero indicates a first fixation on the facial affective picture, but a score less than zero indicates an initial bias away from the facial affective picture. When a score is statistically equal to zero, no bias occurs.
4. Gaze duration bias = total time of fixation on the facial affective picture divided by the total time of fixation on two pictures in the trial. The objective is to test the total attentional fixation. When the score is higher than 50%, it indicates the subject’s total attentional fixation on the facial affective pictures, while a score less than 50% means that the total bias occurs away from the facial affective pictures. When a score is statistically equal to 50%, no bias occurs.

#### Data processing

**Fig 3:**
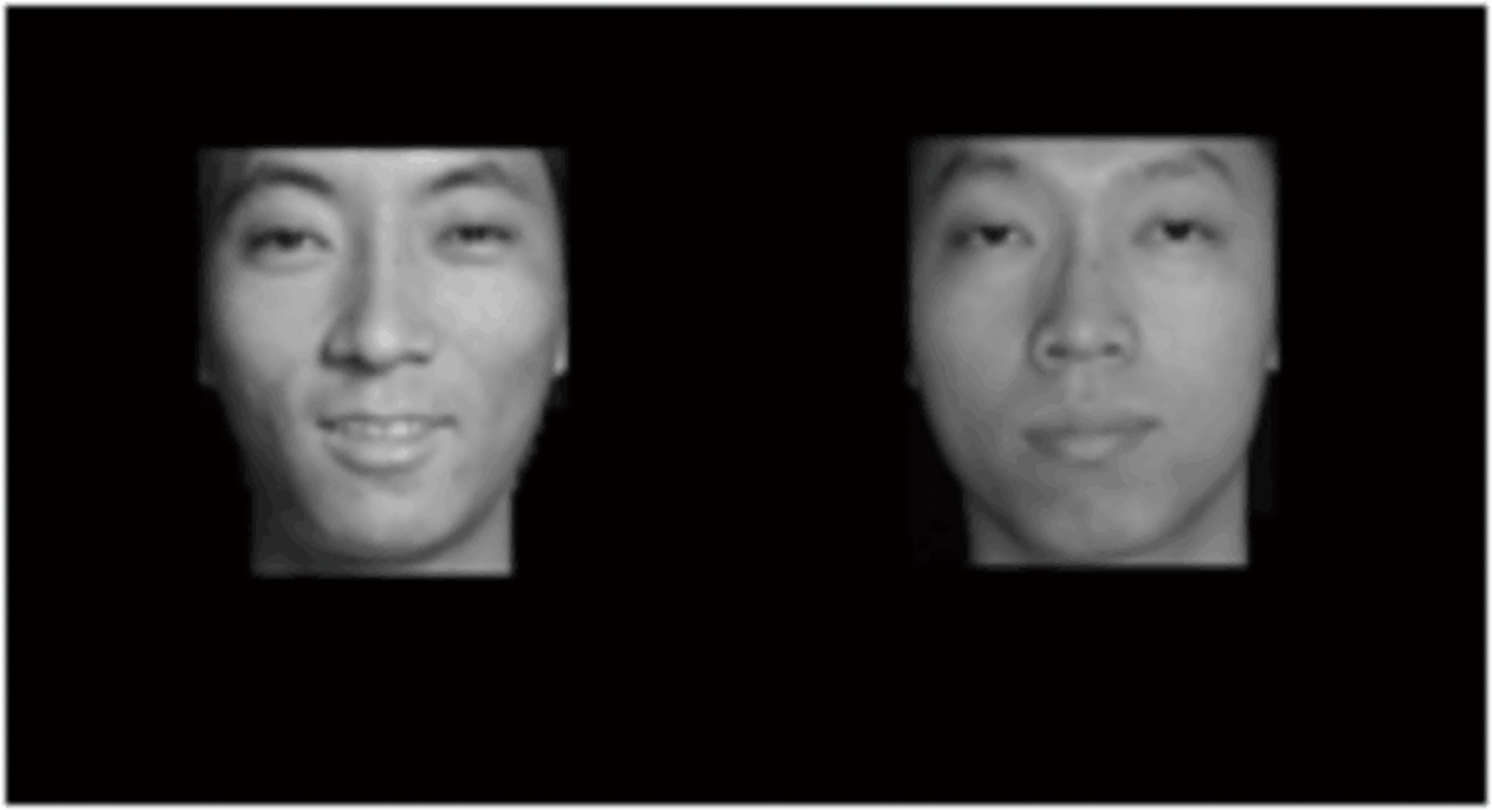
*IAs* of eye tracking in the formal experiment.

Behavioural data and eye-tracking data were processed and screened using Excel 2007, SPSS 16.0, and Data Viewer. Interest areas (IAs) of two picture areas were arranged using Data Viewer. Data that met all of the following conditions were retained in the present study:

1. Before any picture stimulus or a small cross appears, the subject looks attentively at the centre of the screen.
2. The first saccade occurs within 100 ms after the picture stimulus.
3. While the picture stimulus is being presented, the participant’s fixation point is located at either of the two IAs instead of the center of the screen. For data analysis, only that data from within the IAs were retained (Fig 3).
4. The proportion of effective trials is above 50%, and the keying error rate is below 5%. Participants made a keying error when their “F”/ “J” responses did not match the location of the dot probe. The keying error rate was calculated based on the number of times that they made an error in their responses.

A total of 38 participants remained as mentioned before (17 from the social exclusion group and 21 from the control group). For each participant, the trial data with a response time of less than 200 ms or without a timely response (within 1500 ms) were deleted. We did that because a RT less than 200 ms suggested that the participants might have anticipated the stimuli as pointed out by former studies [36,37] and an RT longer than 1500 ms suggests that the participants might have shifted their attention and therefore should be excluded [38]. Only those response times with the right responses were retained, and outliers out of ± 3SD were also deleted. The four eye-tracking indicators described above were collected, including 1) first fixation directional bias score, 2) first fixation latency bias score, 3) first fixation duration bias score, and 4) gaze duration bias score.

The response time bias was calculated by reference to the formula proposed by MacLeod and Mathews [32], namely, response time bias score = [(BrDl—BlDl) + (BlDr—BrDr)] ÷ 2, where **B** denotes the facial affective picture, **D** denotes the probe dot, **l** denotes the left side, and **r** denotes the right side. The response time bias score reflects the difference between the response time when the facial affective picture is opposite to the probe dot and that when they are on the same side. A score higher than zero shows an attentional bias to the facial affective picture, and a score lower than zero indicates an attentional bias away from the facial affective picture; when a score is statistically equal to zero, no bias exists. The response time bias score is used as the total score of the attentional bias.

A 2 (social exclusion group vs. control group) × 2 (P-O vs. N-O facial affective picture pair) ANOVA was adopted to analyse the PANAS, the need to belong, the response time bias score, and the four eye-tracking indicators, with the social interaction condition taken as the between-group variable and the facial affective picture type as the within-group variable. A further simple effect analysis was conducted to determine whether or not the presence of any significant interaction between them could be detected.

## Results

### Scores on the PANAS and the need-to-belong scale

The scores of the PANAS and the NBS were compared between the social exclusion group and the control group (Table 1). The results showed that the positive emotion score of the social exclusion group was significantly lower than that of the control group $\eta_{p}^{2}$. The negative affect score of the social exclusion group was significantly higher than that of the control group. In terms of the need to belong, the score of the social exclusion group was significantly higher than that of the control group. The results indicated a valid manipulation of a social exclusion experience.

**Table 1:** Differences between social interaction conditions (social exclusion group vs. control group) on PANAS and the need to belong.

	Group		*F*	*p*	$\eta_{p}^{2}$
	Social exclusion group (N = 17)	Control group (N = 21)			
**Positive emotions**	22.35 ± 10.53	29.10 ± 9.08	4. 49	. 041	. 11
**Negative emotions**	18.41 ± 4.32	13.71 ± 6.27	6.88	.013	.16
**Need to belong**	48.88 ± 8.54	38.29 ± 9.95	12.07	.001	.25

### Response time

The response times for different picture types (P-O/N-O facial affective picture pair) and the response time bias are reported in Table 2.

**Table 2:** Response time for *P-O* and *N-O* affective pairs in social exclusion and control group (ms).

Location of facial affective picture	Location of probe dot	Social exclusion group			Control group	
		*M*	*SD*		*M*	*SD*
**P-O pairs**						
**Left**	Right	467. 00	88. 97		454. 57	88. 85
**Right**	Right	418. 50	49. 68		443. 64	87. 56
**Right**	Left	445. 32	78. 07		456. 77	83. 38
**Left**	Left	414. 79	65. 93		457. 39	85. 60
**Bias score**		39. 52	52. 68		3. 66	30. 86
**N-O pairs**						
**Left**	Right	454. 80	74. 59		449. 14	79. 86
**Right**	Right	425. 80	60. 09		438. 82	71. 74
**Right**	Left	438. 61	74. 88		450. 94	80. 73
**Left**	Left	418. 96	60. 01		463. 49	97. 63
**Bias score**		24. 32	30. 78		-1. 12	13. 13

We conducted a 2 (between subjects: social exclusion group vs. control group) × 2 (within subject: P-O picture pair vs. N-O picture pair) ANOVA for the response time bias scores. A main effect of social exclusion manipulation existed, *F*(1, 36) = 12.11, *p* = 0.001, $\eta_{p}^{2}$ = 0.25. Specifically, there was a main effect of the manipulation group both for P-O affective pairs, *F*(1, 36) = 6.86, *p* = 0.013, $\eta_{p}^{2}$ = 0.16, and for N-O pairs, *F*(1, 36) = 11.77, *p* = 0.002, $\eta_{p}^{2}$ = 0.25, indicating a longer response time either for P-O or N-O pairs in the social exclusion group than in the control group. The main effect of picture type was not significant, *F*(1, 36) = 2.28, *p* = 0.14, $\eta_{p}^{2}$ = 0.06, and also not within the social exclusion group (*p* = 0.131) or within the control group (*p* = 0.593). The interaction between the condition and picture type was also not significant, *F*(1, 36) = 6.22, *p* = 0.45, $\eta_{p}^{2}$ = 0.02.

The use of a *t*-test showed that for the social exclusion group, the subjects’ response time bias scores were significantly greater than zero when responding both after the positive-neutral facial affective pictures, *t*(16) = 3.09, *p* = 0.007, and after the negative-neutral facial affective pictures, *t*(16) = 3.26, *p* = 0.005, meaning that the subjects produce attentional biases for positive and negative facial affect. However, for the control group, no significant difference existed between the response time bias score and zero when responding after either positive-neutral facial affective pictures, *t*(20) = 0.54, *p* = 0.593, or negative-neutral facial affective pictures, *t*(20) = -0.39, *p* = 0.701.

### Eye-tracking data

Different eye-tracking data for different affective pictures in both conditions are presented in Table 3.

**Table 3:** Four attentional bias indicators for *P-O* and *N-O* affective pairs of social exclusion and control group.

Affective pair type	Affective type	Social exclusion group		Control group	
		*M*	*SD*	*M*	*SD*
**First fixation directional bias (%)**					
**P-O**	**P**	60. 88	7. 34	50. 12	8. 27
**N-O**	**N**	57. 50	10. 31	48. 10	10. 12
**First fixation latency bias (ms)**					
**P-O**	**P**	333. 37	74. 25	341. 13	61. 80
	**O**	372. 26	84. 91	328. 98	74. 39
	**Bias score**	-38. 89	65. 71	12. 15	41. 90
**N-O**	**N**	356. 03	63. 23	352. 30	72. 78
	**O**	359. 29	88. 95	332. 43	77. 00
	**Bias score**	-3. 26	62. 84	19. 87	56. 46
**First fixation duration bias (ms)**					
**P-O**	**P**	304. 13	44. 91	249. 75	65. 54
	**O**	242. 18	59. 64	251. 99	51. 76
	**Bias score**	61. 95	47. 50	-2. 24	53. 50
**N-O**	**N**	272. 56	47. 28	261. 46	45. 17
	**O**	256. 30	69. 87	257. 69	58. 83
	**Bias score**	16. 26	55. 39	3. 77	36. 40
**Gaze duration bias (%)**					
**P-O**	**P**	61. 57	8. 80	49. 83	7. 68
**N-O**	**N**	55. 06	6. 06	49. 77	4. 53

#### (1) First fixation directional bias

A 2 (between subjects: social exclusion vs. control group) × 2 (within subject: P-O vs. N-O facial affective picture pair) ANOVA was conducted on the first fixation directional bias score. The results showed a significant difference between the groups, *F*(1, 36) = 20.68, *p* < 0.001, $\eta_{p}^{2}$ = 0.37. In particular, compared with the control group, the social exclusion group scored significantly higher on the first fixation directional bias both when positive facial picture pairs appeared (*F*(1, 36) = 17.57, *p* < 0.001, $\eta_{p}^{2}$ = 0.33) and when negative facial picture pairs appeared (*F*(1, 36) = 7.98, *p* = 0.008, $\eta_{p}^{2}$ = 0.18). The main effect of picture type was not significant, *F*(1, 36) = 1.86, *p* = 0.181, $\eta_{p}^{2}$ = 0.05, and the interaction was also not significant, *F*(1, 36) = 0.12, *p* = 0.734, $\eta_{p}^{2}$ = 0.003.

A t-test was conducted to check whether, or not, the first fixation directional bias truly existed in the social exclusion group and the control group (compared with 50%, a number indicating a random rate). The results showed that the social exclusion group produced first fixation directional biases (the bias score is greater than 50%) toward the positive facial affective pictures, *t*(16) = 6.11, *p* < 0.001, and toward the negative facial affective pictures, *t*(16) = 3.00, *p* = 0.008; however, the control group did not produce first fixation directional bias toward the positive facial affective pictures, *t*(20) = 0.07, *p* = 0.948, or toward the negative facial affective pictures, *t*(20) = -0.86, *p* = 0.399.

#### (2) First fixation latency bias

A 2 (between subjects: social exclusion vs. control group) × 2 (within subject: P-O vs. N-O facial affective picture pair) ANOVA was conducted on the first fixation latency bias score. A significant main effect of the group on first fixation latency bias as a whole was found, *F*(1, 36) = 7.47, *p* = 0.010, $\eta_{p}^{2}$ = 0.17, indicating a stronger first fixation latency bias of the social exclusion group toward the facial affective pictures than of the control group. The main effect of picture type, although it showed a trend, was not significant, *F*(1,36) = 2.96, *p* = 0.094, $\eta_{p}^{2}$ = 0.08, and the interaction was also not significant, *F*(1,36) = 0.12, *p* = 0.275, $\eta_{p}^{2}$ = 0.03. We further attempted to discover whether the manipulation of social exclusion caused the first fixation latency bias toward both kinds of facial pictures. The results indicated that, compared with the control group, the social exclusion group significantly showed a first fixation latency bias toward the positive facial affective pictures, *F*(1, 36) = 8.45, *p* = 0.006, $\eta_{p}^{2}$ = 0.19, but not toward the negative facial affective pictures, *F*(1, 36) = 1.43, *p* = 0.240, $\eta_{p}^{2}$ = 0.04. In addition, within the social exclusion group, there was a trend that the first fixation latency bias was greater toward the positive facial affective pictures than toward the negative pictures, *F*(1, 36) = 3.62, *p* = 0.065, $\eta_{p}^{2}$ = 0.09, while within the control group, there was no difference in the first fixation latency bias between the positive and negative affective pictures, *F*(1, 36) = 0.21, *p* = 0.650, $\eta_{p}^{2}$ = 0.006.

The results of a direct *t*-test between the first fixation latency bias and zero showed that the social exclusion group performed faster in probing the positive facial affective pictures, *t*(16) = -2.44, *p* = 0.027, with the bias scores less than zero; that is, the social exclusion group’s direct first fixation on the positive facial affective pictures was quicker than on the neutral pictures. No bias was observed in the speed of probing the negative facial affective pictures, *t*(16) = -0.22, *p* = 0.832. For the control group, the speed of probing both the positive facial affective pictures, *t*(20) = 1.33, *p* = 0.198, and the negative facial affective pictures, *t*(20) = 1.61, *p* = 0.123, showed no difference from that for the neutral pictures.

#### (3) First fixation duration bias

As with the former two indicators, we conducted a 2 (between subjects: social exclusion vs. control group) × 2 (within subject: P-O vs. N-O facial affective picture pair) ANOVA to test the first fixation duration bias score. The results showed a significant main effect of the groups, *F*(1, 36) = 13.89, *p* = 0.001, $\eta_{p}^{2}$ = 0.28. The score of the social exclusion group was significantly higher than that of the control group in first fixation duration bias toward the facial affective pictures. The main effect of picture type was not significant, *F*(1, 36) = 2.71, *p* = 0.108, $\eta_{p}^{2}$ = 0.07, but the interaction between group and picture type was significant, *F*(1, 36) = 4.62, *p* = 0.05, $\eta_{p}^{2}$ = 0.11. A further simple effect analysis showed that for the social exclusion group, the positive affect bias score was significantly higher than the negative affect bias score (*F*(1, 36) = 6.52, *p* = 0.015, $\eta_{p}^{2}$ = 0.15), while there was no difference between the positive and negative facial pictures for the control group (*F*(1, 36) = 0.14, *p* = 0.710, $\eta_{p}^{2}$ = 0.004). In addition, the social exclusion group also showed a significantly longer first fixation duration bias than the control group toward the positive facial pictures (*F*(1, 36) = 14.87, *p* < 0.001, $\eta_{p}^{2}$ = 0.29), but the two groups showed no difference toward the negative facial pictures (*F*(1, 36) = 0.69, *p* = 0.411, $\eta_{p}^{2}$ = 0.02).

The results of the *t*-test showed that the social exclusion group performed first fixation directional biases (the bias score was greater than zero) toward the positive facial affective pictures, *t*(16) = 5.37, *p* < 0.001, which means that the social exclusion group kept a longer first fixation on the positive facial affective pictures but not on the negative facial affective pictures when compared with the neutral pictures, *t*(16) = 1. 20, *p* = 0.246. The control group did not have any first fixation on the positive facial affective pictures, *t*(20) = –2.27, *p* = 0.848, or on the negative facial affective pictures, *t(*20) = 3.75, *p* = 0.646.

#### (4) Gaze duration bias

We further conducted a 2 (between subjects: social exclusion vs. control group) × 2 (within subject: P-O vs. N-O facial affective picture pairs) ANOVA to test the gaze duration bias. There was a significant main effect of the group, *F*(1, 36) = 24.47, *p* < 0.001, $\eta_{p}^{2}$ = 0.41. The gaze duration bias score of the social exclusion group toward facial affective picture was significantly higher than that of the control group. The main effect of picture type, *F*(1, 36) = 5.18, *p* = 0.029, $\eta_{p}^{2}$ = 0.13, and the interaction, *F*(1, 36) = 4.96, *p* = 0.032, $\eta_{p}^{2}$ = 0.12, were also significant. A further simple effect analysis shows that the social exclusion group’s positive affect bias score was significantly higher than its negative affect bias score (*F*(1, 36) = 9.18, *p* = 0.005, $\eta_{p}^{2}$ = 0.20), but no significant difference was observed for the control group (*F*(1, 36) = 0.001, *p* = 0.972, $\eta_{p}^{2}$ = 0.001). In addition, the social exclusion group also showed a longer gaze duration bias toward both positive facial pictures (*F*(1, 36) = 19.25, *p* < 0.001, $\eta_{p}^{2}$ = 0.35) and negative facial pictures (*F*(1, 36) = 9.49, *p* = 0.004, $\eta_{p}^{2}$ = 0.21) than the control group.

Furthermore, a *t*-test was used to show that the social exclusion group has a total attentional fixation on the positive facial affective pictures (the bias score was greater than 50%), *t*(16) = 5.37, *p* < 0.001, indicating that the social exclusion group kept the fixation point longer on the positive facial affective pictures but not on the negative facial affective pictures, *t*(16) = 1.2, *p* = 0.246, when compared with that on the neutral pictures. The control group did not exhibit any total attentional fixation bias toward the positive facial affective pictures, *t*(20) = -0.19, *p* = 0.848 or toward the negative facial affective pictures, *t*(20) = 0.47, *p* = 0.642. In this case, although the social exclusion group showed a significant stronger bias toward negative facial picture than the control group, both groups did not show a significant difference with the random rate being 50%. Therefore, the social exclusion actually did not lead to a gaze duration bias toward negative pictures based on this standard.

## Discussion

### Emotional experience, need to belong, and response time bias after social exclusion

The current study indicates that social exclusion has negative consequences for emotional experience and belongingness needs and causes a vigilant state in reacting to social information. The participants in the social exclusion group experienced significantly lower positive emotion, and significantly higher negative emotion, than those in the control group. These findings are consistent with previous studies that argue that social exclusion may induce grief, sadness, indignation, and other negative experiences [5]. In addition, social exclusion also elevated individuals’ need to belong. This finding supports the need-to-belong theory [14], which posits that the belongingness need is one of the fundamental human needs. Moreover, social exclusion experience makes individuals more vigilant to social cues by shortening their reaction time to the probe dot after positive affective or negative affective pictures appear instead of neutral pairs. The general findings that social exclusion causes attentional bias toward both positive and negative facial affective pictures are in line with former studies showing that social exclusion can lead people to seek social cues [26].

The socially excluded individuals in the community tend to show an interpersonal reconnection tendency [6,16]. They are more willing to agree with, or to echo, the other members’ ideas and opinions, and are more willing to coexist with others, to hold optimistic attitudes in potential social relations, and to be generous to those who may indicate friendly social intentions toward them [6]. These behaviours reflect the socially excluded individuals’ psychological desire to be accepted by a group. A lack of a sense of belonging may not only change individuals’ cognitive and attentional processes but also negatively affect their mental health. Individuals who are unable to establish positive social relations may experience depression, guilt, loneliness, and other negative affect [8] and may even suffer serious psychological maladaptive symptoms.

### Attentional biases toward social interpersonal information after social exclusion

Based on the eye-tracking data, the social exclusion group exhibits attentional vigilance to and attentional fixation on social information. Specifically, socially excluded individuals show attentional vigilance to both positive and negative facial affect and total gaze duration on positive facial affect. People are sensitive to facial affect, probably because of our tendency to judge the motives of others through emotional information shown on the face. In human social activities, affective identification is very important. By correctly decoding facial affect, we can quickly and directly access useful social resources, including those who are willing to build social relationships with us and those who might help us when we need assistance. Affective identification is indeed a skill that reflects the development of individuals’ social abilities [39].

Individuals show vigilance to and attentional fixation on the positive affective pictures, including higher first fixation directional bias, stronger first fixation latency bias, first fixation duration bias, and longer gaze duration for P-O pictures in the social exclusion group than in the control group. Meanwhile, there is also stronger first fixation directional bias toward the negative facial affective pictures in social exclusion group than the control group. The findings reveal that the underlying motives toward the positive and the negative affect pictures might be different. Social exclusion makes individuals seek new social relationships and become more sensitive to avoiding negative social information. This could be a reason why the social exclusion group showed significant higher first fixation directional bias toward negative affective pictures than the control group, but did not show first fixation duration bias and gaze duration bias toward negative affective pictures. However, the motive to affiliate and connect with others might generate the longer gaze duration on the positive social cues. The results are consistent with former research, which indicates that social exclusion lead to selective attention to positive affective information and an attunement to social acceptance cues [26]. More than that, although there is no fixation duration bias toward negative affective pictures, this study reveals the vigilance toward negative cues, namely first fixation directional bias, which is not presented in other earlier research.

The current study contributes to the literature in several ways. First, it provides objective and integral evidence based on eye movement that social exclusion increases individuals’ attentional bias toward social cues, especially positive social cues. Compared with former studies of attentional bias after social exclusion, such as the study undertaken by DeWall and colleagues [26], the current study provides more integral results that cover four eye-movement indicators from first fixation directional bias, first fixation latency bias, first fixation duration bias, to gaze duration bias. It reveals a significant first fixation directional bias toward the negative affective pictures, which the DeWall group did not report. Second, because social exclusion attracts attention from researchers and workers in many domains, including clinical psychologists, social workers, school educators and parents[40,41], this study sheds light on individuals’ reactions after social exclusion. Former studies found that social exclusion caused reactions of the physical pain system or experiences similar to physical pain [3,42]. We provide evidence through attentional bias indicators that social exclusion causes individuals to seek social cues out of the motive, and need, to belong. A way to start to reduce painful experience, and the related negative impacts, of social exclusion is to provide positive and supportive social information to those who are socially excluded.

### Limitations and future research directions

Although our hypotheses are supported in the current study, there are still some limitations. First, while we measured the participants’ positive and negative emotions in the pilot study and the need to belong in the formal experiment, a direct manipulation check of the sense of social exclusion is not included. We suggest that, in future research, a direct manipulation check of social exclusion (*e*.*g*., the need-threat scale; see van Beest & Williams[29]) be included to indicate the validation of the social exclusion manipulation. Second, the eye-tracking experiment lasted for a relatively long period that might have caused impatience and negative affect among the participants. Future studies might consider shortening the period of the task to an extent that would make the experiment more efficient. Third, we only used P-O, N-O, and O-O pairs of affective pictures, but did not include P-N pictures. We suggest that a direct comparison between positive and negative affective pictures by pairing them would provide extra information about individuals’ attentional bias toward social cues after social exclusion. In addition, the current study does not include individual differences in traits as moderators, which might also play a role in the process. For example, self-esteem is viewed as an interpersonal monitor [43] and plays an important role in social interaction. Those with low self-esteem would pursue more interpersonal information, while those with high self-esteem might be more likely to seek information that reflects their ability or competence [44]. Therefore, it is possible that those with low self-esteem might exhibit stronger attentional bias toward social cues after social exclusion. Future studies might include some moderators that would help to pinpoint what kinds of people are more influenced by social exclusion.

## Conclusions

In conclusion, this study shows that social exclusion causes a more negative, and less positive, emotional experience, a higher need to belong, and a longer response time in the probe dot task. It also causes attentional bias toward positive and negative facial affective information. Individuals, after social exclusion, show initial directional bias, first fixation latency bias, first fixation duration bias, and greater total attentional fixation duration toward positive facial affective pictures but show directional bias only toward negative facial affective pictures. This means that socially excluded individuals’ positive affective response time bias score exhibits attentional vigilance and attentional fixation toward positive affective pictures.

Nevertheless, the current research demonstrates the emotional and attentional consequences of social exclusion through a self-report measure and use of an eye-tracking technique and hence is valuable in providing a deeper understanding of social exclusion.

## Supporting information

S1 SAV Data revision. (SAV)
